# Exploration of Exopolysaccharide from *Leuconostoc mesenteroides* HDE-8: Unveiling Structure, Bioactivity, and Food Industry Applications

**DOI:** 10.3390/polym16070954

**Published:** 2024-03-30

**Authors:** Yi Yang, Guangbin Ye, Xintong Qi, Bosen Zhou, Liansheng Yu, Gang Song, Renpeng Du

**Affiliations:** 1 Engineering Research Center of Agricultural Microbiology Technology, Ministry of Education & Heilongjiang Provincial Key Laboratory of Plant Genetic Engineering and Biological Fermentation Engineering for Cold Region & Key Laboratory of Microbiology, College of Heilongjiang Province & School of Life Sciences, Heilongjiang University, Harbin 150080, China; 2221595@s.hlju.edu.cn (Y.Y.); 20224158@s.hlju.edu.cn (X.Q.); 2221609@s.hlju.edu.cn (B.Z.); 2211628@s.hlju.edu.cn (L.Y.); 2 Institute of Life Sciences, Youjiang Medical University for Nationalities, Baise 533000, China; ygb9064@ymun.edu.cn; 3 Hebei Key Laboratory of Agroecological Safety, Hebei University of Environmental Engineering, Qinhuangdao 066102, China; 4 State Key Laboratory of Microbial Metabolism, School of Life Sciences and Biotechnology, Shanghai Jiao Tong University, Shanghai 200240, China

**Keywords:** *Leuconostoc mesenteroides*, exopolysaccharide, structure, physicochemical, food application

## Abstract

A strain of *Leuconostoc mesenteroides* HDE-8 was isolated from homemade longan fermentation broth. The exopolysaccharide (EPS) yield of the strain was 25.1 g/L. The EPS was isolated and purified, and the structure was characterized using various techniques, including X-ray diffraction (XRD), nuclear magnetic resonance (NMR) spectroscopy, Fourier-transform infrared (FT-IR) spectroscopy, high-performance size exclusion chromatography (HPSEC), and scanning electron microscopy (SEM). The monosaccharide composition of the EPS was glucose, with a molecular weight (Mw) of 1.7 × 10^6^ Da. NMR spectroscopy revealed that the composition of the HDE-8 EPS consisted of *D*-glucose pyranose linked by α-(1→4) and α-(1→6) bonds. The SEM analysis of the EPS showed an irregular sheet-like structure. Physicochemical analysis demonstrated that EPSs exhibit excellent thermal stability and high viscosity, making them suitable for fermentation in heat-processed and acidic foods. Additionally, milk coagulation tests showed that the presence of EPSs promotes milk coagulation when supplemented with sucrose. It suggests that EPSs have wide-ranging potential applications as food additives, improving the texture and taste of dairy products. This study provides practical guidance for the commercial use of HDE-8 EPSs in the food and related industries.

## 1. Introduction

Exopolysaccharides (EPSs) are produced by bacteria, yeasts, fungi, and cyanobacteria and are widely distributed in nature [1]. EPSs can be classified into homopolysaccharides and heteropolysaccharides based on the monomeric composition of the polysaccharide chains. Homopolysaccharides are composed of a single type of sugar monomer and are named based on the monosaccharide type and the anomeric configuration between individual units. For instance, homopolysaccharides derived from glucose can be categorized as α-glucan or β-glucans. Heteropolysaccharides contain two or more different types of monosaccharides, and the specific arrangement and combination of these monosaccharides in heteropolysaccharides give rise to diverse structures [2]. Research has demonstrated that the properties of EPSs are influenced not only by their chemical structure, but also by their composition, surface morphology, and glycosidic linkages. These characteristics make EPS a favorable option for numerous industrial applications [3,4,5].

Lactic acid bacteria (LAB) are a common type of bacteria known for their good safety profile and generally recognized as safe (GRAS) [3]. EPSs have emerged as a prominent research focus, garnering considerable attention from experts. As a secondary metabolite of LAB, it is a naturally occurring polymer known for its anti-tumor, antioxidant, and biodegradable properties. Beyond its environmentally friendly characteristics, EPSs exhibit a diverse range of functionalities, making them a subject of widespread interest in the realm of biological sciences [6,7,8,9]. EPSs produced by LAB have received significant attention due to their potential use as a natural food ingredient or additive, replacing chemical reagents in the processing of health and functional foods [10,11]. Research has found that LAB capable of producing EPSs include *Lactobacillus*, *Lactococcus*, *Leuconostoc*, *Pediococcus*, and *Streptococcus* [12,13]. The structure and function of some LAB EPSs have been reported in previous studies. For instance, *Lactobacillus reuteri* E81 produces α-glucan that contains α-(1→3) and α-(1→6) glycosidic linkages. The water holding capacity and heat resistance of these EPSs make them suitable for food applications [14]. Other research has shown that EPSs produced by LAB play a role in thickening, improving texture, and enhancing stability in dairy products [15]. Similarly, in baked products, LAB that synthesize EPSs are used to enhance product texture, sensory characteristics, and storage stability. EPSs also benefit baked goods by forming a network with water and dough components, improving the rheology, bulk, and size of bread and extending its shelf life [16,17]. Therefore, incorporating EPSs produced by LAB as natural food ingredients or additives in the production of health and functional foods can serve as a viable alternative to chemical reagents, thereby improving food safety and quality.

The current yield of EPSs produced by the fermentation of LAB is still low, and the structure and properties of EPSs from different sources of LAB vary considerably. Furthermore, the relationship between structure and function has not been fully explained. In order to expand the number of high-yield EPS-producing strains, characterize the structure and biochemical characteristics of EPSs, and explore their potential applications in the food industry, the objective of this study is to isolate a high EPS-producing strain from longan fermentation broth and to determine the structure and physicochemical properties of the EPS produced by this strain.

## 2. Materials and Methods

### 2.1. Isolation and Identification of Strains

The longan sample from Fujian, China, was used to isolate the candidate bacterial strains that produce EPSs. Firstly, the samples were serially diluted, then the dilutions were applied to MRS-S solid medium and then incubated at 30 °C for 48 h. The formation of mucoid substances secreted by the candidate strains after incubation was observed, indicating that an EPS-positive signal was generated. Then, single stripe strains showing an EPS-positive signal were selected from MRS medium. The genomic DNA of candidate strain was isolated with genomic DNA isolation kits (Tiangen Biotech Co. Ltd., Beijing, China). PCR amplification was performed using primers (8F:5′-AGAGTTTGATCATGGCTCAG-3′ and 1492R: 5′-ACGGTTACCTTGTTACGACTT-3′), and then PCR products were sequenced. The sequencing results can be used to analyze the genome and submit it to the NCBI database. The phylogenetic tree was constructed using the neighbor-joining (NJ) method [18].

### 2.2. Isolation and Purification of HDE-8 EPS

The HDE-8 strain was inoculated into 250 mL of MRS-S liquid medium and cultured at 30 °C for 48 h at 120 rpm. Subsequently, the separation and purification of EPS were carried out according to the method described by Du et al. [18], and pure freeze-dried EPS was obtained in the end. The HDE-8 EPS solution was prepared at a concentration of 1 mg/mL using deionized water. The purity of HDE-8 EPS was measured using a UV-visible spectrophotometer (UV-2550, Shimadzu, Kyoto, Japan) in the wavelength range of 190–400 nm. The carbohydrate content was measured by the phenol–sulfuric acid method [19]. The protein content of HDE-8 EPS was determined by the Bradford method [20]. The glucuronic acid content was measured by the sulfuric acid–carbazole method [21]. The sulfate group content was determined by the barium chloride–gelatin method [22].

### 2.3. Monosaccharide Composition Analysis of HDE-8 EPS

The monosaccharide composition of EPS was analyzed according to previously reported methods [18]. A total of 2 mg of HDE-8 EPS was dissolved in anhydrous methanol containing 1 M hydrochloric acid and incubated for 16 h at 80 °C followed by the addition of 2 M TCA and incubation for 1 h at 120 °C. The sample was derivatized using 1-methoxy-2-propyl propionate. The monosaccharide composition of the HDE-8 EPS sample was analyzed using high-performance liquid chromatography (HPLC, LC-20AT, Shimadzu, Kyoto, Japan).

### 2.4. Molecular Weight (Mw) Distribution

For the Mw analysis of HDE-8 EPS, a previously described methodology was used [23]. The Mw determination of the HDE-8 EPS sample was carried out using high-performance size exclusion chromatography (HPSEC, DGU-14A, Shimadzu, Kyoto, Japan). A specialized column, Shodex OH-park SB-805 (8.0 × 300 mm), and a refractive index detector were used. Prior to injection into the HPSEC system, both the dextran standards and EPS sample were filtered through a 0.2 μm membrane filter at a concentration of 2 mg/mL. The analysis by chromatography was performed at a temperature of 30 °C and a flow rate of 0.8 mL/min. Dextran standards (1170, 1740, 2400, and 3755 kDa) were used to establish a standard curve for calculating the Mw of samples.

### 2.5. Fourier-Transform Infrared (FT-IR) Spectroscopy Analysis

The FT-IR spectra of HDE-8 EPS was collected using BIO-RAD IR spectrometer (FTS3000, Bruker, Karlsruhe, Germany). KBr discs were used as the sample matrix for analysis, with spectra recorded in the 400–4000 cm^−1^ range. To enhance the signal-to-noise ratio, 32 scans were accumulated for each spectrum [19].

### 2.6. Nuclear Magnetic Resonance (NMR) Spectroscopy Analysis

NMR analysis was used in this study to explore the structural properties of HDE-8 EPS. The purified EPS sample (30–50 mg) was subjected to three exchanges with deuterated water (D_2_O) to remove any residual solvent containing protons. After the exchange, the EPS sample was dissolved in 0.55 mL of D_2_O and transferred to an NMR tube for NMR spectroscopy analysis using an Avance III NMR spectrometer (Avance III, Bruker, Germany). The NMR experiments conducted included ^1^H NMR, ^13^C NMR, HSQC, and COSY. The obtained NMR data were processed and analyzed using MestReNova software (MestReNova x64-14.2.1, Mestrelab Research, Santiago de Compostela, Spain) [24].

### 2.7. Scanning Electron Microscopy (SEM) and Atomic Force Microscopy (AFM) Analysis

According to the previously reported method, SEM and AFM were used to detect the surface morphology of the HDE-8 EPS sample [3]. The EPS samples were observed at an accelerating voltage of 2 kV using SEM (S4800, Hitachi, Tokyo, Japan) and images were obtained at 350× and 1000× magnifications. The AFM images were acquired using a scanning probe microscope (Dimension ICON, Bruker, Karlsruhe, Germany).

### 2.8. X-ray Diffraction (XRD) Analysis

XRD analysis was conducted to determine the crystal structure of HDE-8 EPS. The XRD pattern was obtained using an X-ray powder diffractometer (X’Pert PRO MPD, Panalytical, Almelo, The Netherlands) in the 2θ range of 10° to 80°, with a scanning rate of 2°/min [19].

### 2.9. Thermal Properties Analysis

Thermogravimetric analysis (TGA), differential thermogravimetry (DTG), and differential scanning calorimetry (DSC) were performed on the Maia F3 200 device (Netzsch, Selb, Germany). Then, 10 mg HDE-8 EPS sample was heated in a nitrogen atmosphere at a linear heating rate of 10 °C/min from 40 °C to 800 °C, using an Al_2_O_3_ crucible [14].

### 2.10. Water Contact Angle Measurement

The water contact angle was determined using the method described by Du et al. [25]. HDE-8 strain was cultured in two different media: MRS and MRS + 5% (*w*/*v*) sucrose for 48 h. After the fermentation period, the broth culture was subjected to centrifugation, and then the supernatant was filtered through a 0.45 μm filter, and the filter was subsequently dried at room temperature. The water contact angle of the dried filters was measured using a contact angle analyzer (LSA50, LAUDA Scientific, Brandenburg, Germany).

### 2.11. Milk Coagulation Test

The HDE-8 strain was pre-cultivated in MRS medium with a 1% (*v*/*v*) inoculum. It was then mixed with 10% (*w*/*v*) sterile skim milk and 0, 3, or 6% (*w*/*v*) sucrose. The mixture was incubated at 37 °C for 48 h to perform a solidification test. A negative control was used, consisting of a mixture of the HDE-8 strain and skim milk without any added sucrose [4].

### 2.12. Emulsifying Activity (EA) Assay

Following the method of Du et al. [26], 2.5 mL of the purified HDE-8 EPS solution (1.0 mg/mL) was mixed with 2.5 mL of hydrocarbons or oils (hexane, benzene, xylene, petroleum ether, ether, gasoline, diesel oil, or soybean oil). The mixed liquor was injected in a glass tube and placed at 25 °C for 72 h. The EA was calculated by Equation (1):

EA (%) = (M_1_/M_0_) × 100 (1)

where M_0_ is the full height of mixture and M_1_ is the height of emulsifying layer.

### 2.13. Heavy-Metal-Chelating Activity

The heavy metal chelating performance of HDE-8 EPS was determined using an atomic absorption spectrometer (iCE 3500, Thermo Scientific, Waltham, MA, USA), according to the method described by Du et al. [25]. In this experiment, 2 mL of HDE-8 EPS solution (10 mg/mL) was mixed with a 50 mL solution containing 10 mg/L of the metals Cu^2+^, Fe^2+^, Zn^2+^, Pb^2+^, and Cd^2+^. The mixture was incubated at room temperature for 2 h and centrifuged at 10,000 rpm for 10 min.

### 2.14. Rheological Properties

The rheological properties of the HDE-8 EPS were measured according to the method described by Wang et al. [27]. A rheometer (HAAKE MARS60, Karlsruhe, Germany) was used to measure the viscosity of a 2% (*w*/*v*) EPS solution at different pH levels (4, 6, and 8) and temperatures (25, 30, 35, 40, and 45 °C). The rotational speeds used for the measurements were 0.6 and 60 1/s. In order to assess the effect of metal ion solutions on HDE-8 EPS, EPS solutions were prepared using 0.1 M CaCl_2_ and NaCl. The viscosity of these solutions was also measured using the rheometer.

### 2.15. Statistical Analysis

The experimental data were reported as mean ± standard deviation. Statistical analysis was carried out using JMP (version 9.0.2, SAS Institute Inc., Cary, NC, USA) software, with a significance level set at *p* < 0.05 for one-way analysis of variance (ANOVA). Additional statistical analyses were performed using Origin 9.0 software (OriginLab Corporation, Northampton, MA, USA).

## 3. Results and Discussion

### 3.1. Isolation and Identification of EPS-Producing Strain

**Figure 1:**
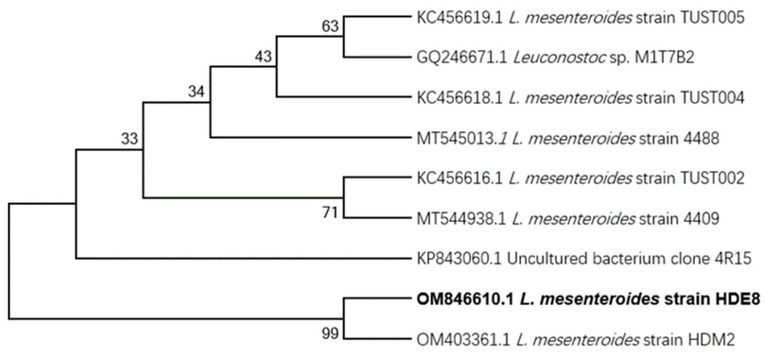
NJ tree representing the phylogenetic relationship based on 16 s rDNA gene sequences.

The strain HDE-8 isolated from Fujian longan fermentation broth-produced EPSs and its colonies were slightly convex, with regular edges, slime, and a cream color on the MRS-S medium. Based on morphological observation and 16S rDNA sequence analysis, the similarity between the HDE-8 strain and the *L. mesenteroides* HDM2 strain reached 99%. By constructing an NJ phylogenetic tree (Figure 1), the relationship between the HDE-9 strain and its close relatives was determined. This bacterium has been identified as *L. mesenteroides* (GenBank accession number OM846610.1). The EPS production of the HDE-8 strain at 48 h was 25.1 g/L, which was higher than the EPS production of *Leuconostoc pseudomesenteroides* YF32 (12.5 g/L) [28], but lower than that of *L. pseudomesenteroides* DRP-5 (43.1 g/L) [26].

### 3.2. Purification and Chemical Composition of HDE-8 EPS

**Figure 2:**
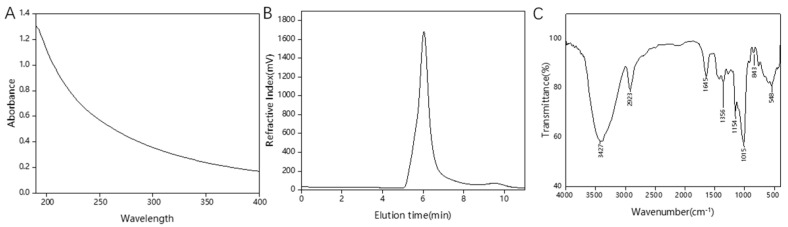
UV–vis spectrum (**A**), HPSEC (**B**) chromatogram, and FT-IR spectrum (**C**) of the purified HDE-8 EPS.

The crude HDE-8 EPS produced a symmetric peak with a Sephadex G-100 gel filtration column, indicating a uniform composition. Upon freeze-drying, the purified HDE-8 EPS obtained was a white fluffy solid. UV-Visible spectroscopy analysis (Figure 2A) showed no absorption peaks at 260 nm and 280 nm, suggesting that the HDE-8 EPS was free from protein and nucleic acid. Chemical composition analysis shown that the HDE-8 EPS only contains neutral carbohydrates, with no detected content of proteins, sulfates, and uronic acids.

### 3.3. Monosaccharide Composition and Mw Analysis

The purified HDE-8 EPS was found to be a homopolysaccharide. This was confirmed by the analysis of the monosaccharide composition. By comparing retention times with various standard monosaccharides, it was established that the HDE-8 EPS predominantly consists of glucose monosaccharide units. Consequently, the HDE-8 EPS can be characterized as a glucan comprising glucose units. 

In the Mw results of the EPS, depicted in Figure 2B, a distinct and symmetrical narrow peak was observed in the HPSEC chromatogram, confirming the homogeneity of the EPS. The Mw of the EPS was calculated as 1.7 × 10^6^ Da according to the standard curve. In general, the Mw of LAB EPSs was in the range of 4.0 × 10^4^ Da to 6.0 × 10^6^ Da [29]. The Mw of HDE-8 EPSs was lower than that of EPSs from other strains, such as the EPSs from *L. pseudomesenteroides* DRP-5 composed of glucose (6.2 × 10^6^ Da) [26], but higher than that of *Weissella cibaria* GA44 composed of glucose and rhamnose (2.2 × 10^5^ Da) [30] and *W. cibaria* JAG8 composed of glucose (8.0 × 10^5^ Da) [31]. The Mw plays a significant role in determining the bioactivity of EPSs. Research has found that the difference in Mw affects the properties of LAB EPSs, including their viscosity, flocculation, emulsifying properties, solubility, and antioxidant performance [32]. When the Mw of an EPS is high, the EPS solution is more viscous and has better flocculation activity; when the Mw of an EPS is low, the solubility is higher and it has better antioxidant activity [33]. The higher Mw of HDE-8 EPSs have a better viscosity and may be suitable for use as a thickener in the food industry.

### 3.4. FT-IR Analysis

The functional groups and glycosidic linkages of HDE-8 EPSs were identified using FT-IR analysis. The FT-IR spectra of HDE-8 EPSs (Figure 2C) exhibited characteristic absorption peaks of polysaccharides, suggesting that the HDE-8 EPS was a carbohydrate. At 3427 cm^−1^, the broad stretching peak observed in the HDE-8 EPS was attributed to the stretching vibration of the hydroxyl groups (O-H) present in the EPSs [34]. The band at around 2923 cm^−1^ was associated with the C-H stretching vibration of the polysaccharide. The peak at 1645 cm^−1^ was a result of bound water [35]. The fingerprint region, spanning from 1200 to 800 cm^−1^, was utilized for characterizing various polysaccharides [36]. Additionally, the peak at 1015 cm^−1^ indicates the significant chain flexibility near the α-(1→6) linkages in EPSs. The weak peak at 843 cm^−1^ was characteristic of α-anomers. The skeletal form of the pyranose ring was responsible for the bending vibration observed at 548 cm^−1^. 

### 3.5. NMR Analysis

**Figure 3:**
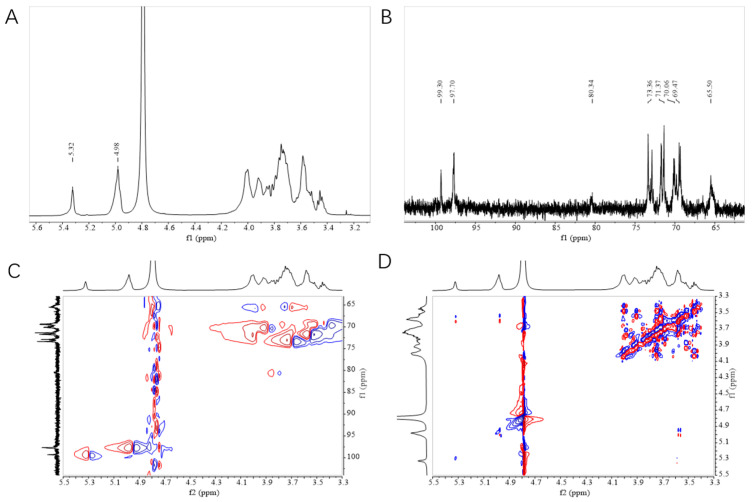
^1^H (**A**), ^13^C (**B**), HSQC (**C**), and COSY (**D**) NMR spectrum of HDE-8 EPS.

The ^1^H NMR (Figure 3A), ^13^C NMR (Figure 3B), HSQC (Figure 3C), and COSY (Figure 3D) NMR spectroscopy were used to analyze the type and configuration of glycosidic bonds in the structure of HDE-8 EPSs. The ^1^H NMR spectrum showed peaks in the region of 3.2 ppm and 5.5 ppm. Anomeric signals appeared at approximately δ5.32 ppm and 4.98 ppm, corresponding to the H-1 of α-(1→4)- and α-(1→6)-linked *D*-glucose residues [37]. Importantly, the high intensity of the H-4 signal (close to 3.45 ppm) in the terminal glucose residues indicated a high level of branching [38]. The ^13^C NMR spectroscopy of the HDE-8 EPSs confirmed the ^1^H data, with the major carbon forms in the anomeric region located at δ99.30 and 97.70 ppm, designated as α-(1→4) and α-(1→6) linkages [39]. Four anomeric peaks observed at δ73.36, 71.37, 70.06, and 69.47 ppm represented glucose residues substituted at C-3, C-2, C-5, and C-4. The signal at δ65.50 ppm corresponds to the C-6 carbon of the glucose unit, suggesting that the two glucose units in the glucose chain backbone are predominantly linked by α-(1→6) connections [40]. The major resonance in the hetero region occurred at δ97.70 ppm instead of 90 ppm, indicating the presence of a C-1 linkage. Similarly, a signal of equal intensity was observed at δ65.50 ppm instead of 60 ppm, indicating that most of the C-6 was also connected [41]. The absence of peaks between δ101 and 105 ppm in the ^13^C NMR spectrum indicated that the EPSs consisted only of α-glucosidic bonds. A peak of the same intensity was present at δ80.34 ppm, indicating the presence of branch connections and a pyranose ring configuration in the EPS [42]. 

The correlation between the proton signals observed in the COSY and HSQC spectra of the HDE-8 EPS proved the presence of a single sugar residue in the EPS repeating unit. In the anomeric region, the C-1 signal at δ99.30 and 97.70 ppm were connected to the proton signals at δ5.32 and 4.98 ppm. The proton signals (5.32/99.30 (H1′/C1′), 4.98/97.70 (H1/C1), 3.58/71.37 (H2/C2), 3.66/73.36 (H3/C3), 3.52/69.47 (H4/C4), 3.86/70.06 (H5/C5), and 3.75, 3.93/65.50 (H6, H6′/C6)) were observed in the HSQC spectrum, verifying the existence of α-(1→4) and α-(1→6) glucose residues within the glucose repeating unit of HDE-8 EPSs. This was consistent with the EPSs synthesized by *L. reuteri* SK24.003, which are composed of α-(1→4)- and α-(1→6)-linked *D*-glucose pyranose [43].

### 3.6. SEM and AFM Analysis

**Figure 4:**
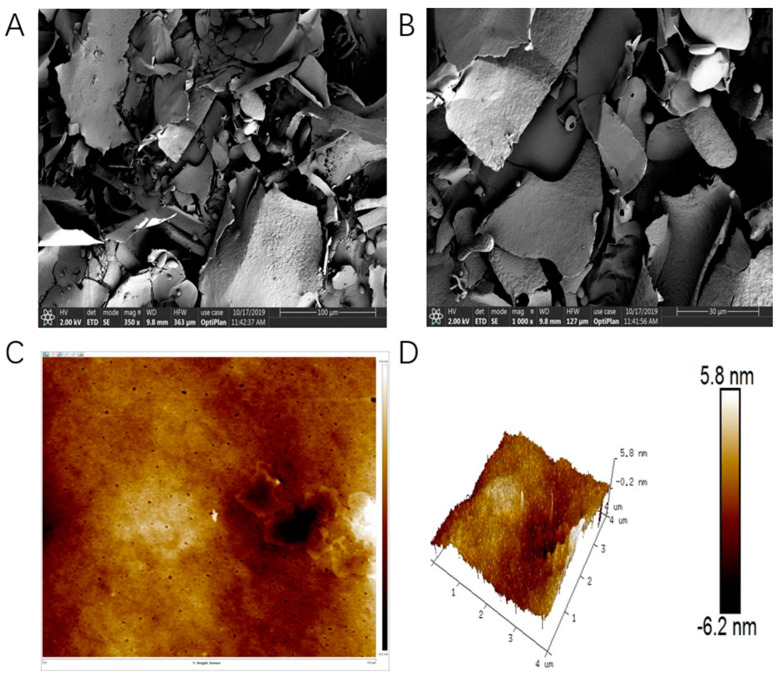
SEM images of EPS at 350× (**A**) and 1000× (**B**) magnification. AFM images of HDE-8 EPS, planar view (**C**) and cubic view (**D**).

The application of SEM technology reveals the surface and microstructure of EPS, providing us with detailed and clear images. As shown in Figure 4A,B, irregular sheets and branching structures were found in the micrographs of the microstructure of the HDE-8 EPS, which could better improve the rheological properties of food [44]. At 1000× magnification, the smoother and denser surface of the HDE-8 EPS made it a high-quality material for food packaging films. Prior research indicates that smooth surface EPSs can enhance the water solubility, water retention capacity, and viscosity of the product [18].

The surface morphology, structure, and surface roughness can be effectively characterized using AFM, making it a powerful tool for studying the physical properties, morphological characteristics, and dynamics of EPSs. AFM enables the visualization and analysis of nanoscale features and interactions, offering valuable insights into the structure–function relationships of EPSs. The AFM image of the HDE-8 EPS is shown in Figure 4C,D. The surface of the HDE-8 EPS was uneven and rough, with block-like and columnar irregular protrusions with a height of −6.2–5.8 nm. These irregular blocks may be caused by the aggregation of EPS macromolecules. EPS macromolecules demonstrate a high affinity for water molecules and display pseudoplastic characteristics. The findings of this study align with earlier observations that *Leuconostoc lactis* L2 EPSs possess a blocky surface structure and demonstrate both an affinity for and pseudoplastic behavior towards water molecules [23].

### 3.7. XRD Analysis

**Figure 5:**
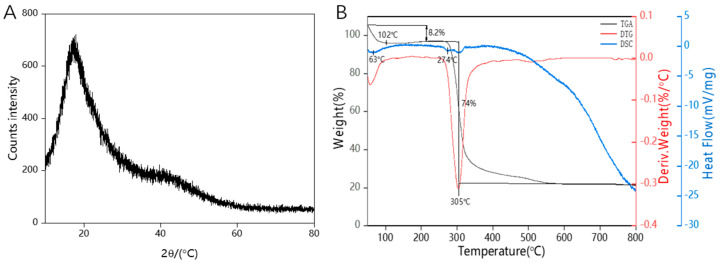
The XRD spectra (**A**) and thermal curves (**B**) of HDE-8 EPS.

As shown in Figure 5A, there was a broad peak around 20° (2θ) in the XRD spectrum, indicating the non-crystalline amorphous nature of the EPS. In XRD patterns, amorphous materials typically appear as broad peaks rather than sharp diffraction peaks. This was because the molecular arrangement in the amorphous materials lacks regular periodicity, resulting in a wide distribution of scattering angles and intensities. Additionally, consistent with other studies, EPSs produced by *Lactobacillus kefiri* also exhibit similar amorphous characteristics [45]. Therefore, the XRD results indicate the amorphous nature of the EPS, characterized by the presence of a broad peak around 20° (2θ). These findings were significant for understanding the structure and properties of the EPS and providing a foundation for its potential applications in biomedical and material science.

### 3.8. Thermal Properties Analysis

The performance of the HDE-8 EPS in terms of thermal characteristics is depicted in Figure 5B. The TGA curve effectively illustrates how the HDE-8 EPS experiences a decline in mass as the temperature gradually rises from 40 °C to 800 °C. The degradation of the HDE-8 EPS occurs in three main stages. The first stage, which occurs between 40 °C and 102 °C, results in a mass loss of approximately 8.2%, mainly due to the loss of moisture in the EPS [46]. In the second stage, the EPS mass remains constant between 102 °C and 274 °C, indicating that the EPS is stable in this temperature range. Finally, a maximum mass loss of approximately 74% is observed between 274 °C and approximately 708 °C, which may be due to the depolymerization of the HDE-8 EPS [47]. The DTG curve shows that the thermal decomposition temperature of the HDE-8 EPS is relatively high, with a degradation temperature of 305 °C, which is higher than the thermal decomposition temperature of commercial xanthan gum (294 °C) [48]. Finally, as the temperature increases, the mass of the sample remains constant. This is due to the presence of complex molecular structures, such as sugar aldehydes in HDE-8 EPSs. The changes in heat absorption and the release of the sample with the increasing temperature are analyzed from the DSC curve. There are three endothermic peaks in the DSC curve for HDE-8 EPSs. The initial peak (63 °C) is primarily attributed to the sample’s moisture evaporation [49]. The second melting endothermic peak (274 °C) indicates the transition between the melting and crystallization phases of the sample [4]. The high thermal stability of the thermal decomposition reaction of the HDE-8 EPS is suggested by the third endothermic peak at 305 °C. In conclusion, the HDE-8 EPS has good thermal stability, which can be compounded with other materials to make composite films, which are widely used in the field of food packaging.

### 3.9. Water Contact Angle Analysis

**Figure 6:**
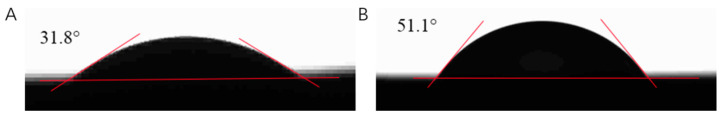
Water contact angle analysis of HDE-8 EPS in MRS at 17 s (**A**) and MRS +5% sucrose at 17 s (**B**).

The water contact angle is an important parameter related to surface hydrophobicity, where a smaller contact angle indicates better hydrophilicity [50]. The results, as shown in Figure 6, revealed a contact angle of 31.8° for the MRS medium and 51.1° for the MRS medium with 5% sucrose. The addition of sucrose induces *L. mesenteroides* HDE-8 to produce EPSs, thereby increasing its surface hydrophobicity, which was similar to the characteristics of the previously reported *W. cibaria* 27 EPS [50]. The study also found that the contact angle of LAB is related to the structure of surface layer proteins [18]. Therefore, it is speculated that the production of EPSs by HDE-8 leads to changes in its structure, resulting in variations in its hydrophobicity and contact angle.

### 3.10. Milk Solidification Analysis

**Figure 7:**
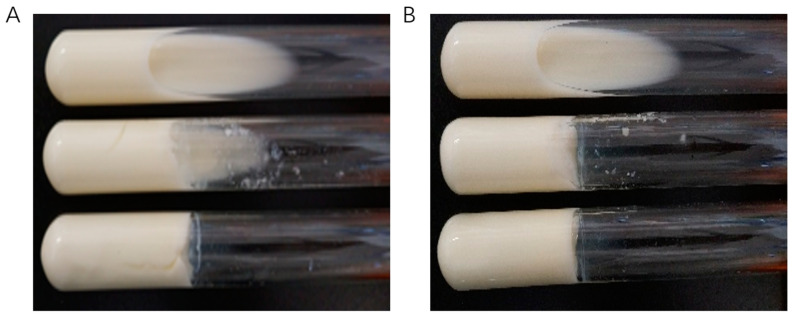
Solidification of 10% skim milk in the mixture of 36 h (**A**) and 48 h (**B**) pre-cultivated *L. mesenteroides* HDE-8. In each diagram, the concentration of sucrose in the tubes from top to bottom was 0, 3, and 6%, respectively.

One study found that adding sucrose can promote the production of EPS by LAB, thereby improving the coagulation effect of 10% skim milk [51]. Figure 7 shown the coagulation effect of *L. mesenteroides* HDE-8 on skim milk. The control sample of skim milk (0% sucrose) did not solidify. However, the coagulation effect of the experimental group increased with the sucrose concentration and fermentation time. When the sucrose concentration was 6%, skim milk was almost completely coagulated, indicating that the presence of sucrose promoted the production of EPSs. Previous research has indicated that the coagulation of skim milk may be due to the interaction between EPSs and proteins in skim milk. This interaction is related to the structure and Mw of EPSs, the type of proteins in skim milk, and the ratio of EPSs to milk proteins [52]. This study presents a theoretical foundation for the production of solid dairy products.

### 3.11. EA Activity

Emulsifiers help stabilize two immiscible liquids, allowing them to be uniformly dispersed together. Extensively employed in the chemical, food, pharmaceutical, and petroleum sectors, microbial emulsifiers have garnered significant interest for their notable attributes of high biodegradability and low toxicity. The functional groups present in the EPS molecular chain play a crucial role in determining the emulsification efficacy [53]. Table 1 illustrated that the emulsifying capacity of HDE-8 EPSs with organic solvents escalates over time. At 72 h, the emulsification rate was successively soybean oil > petroleum ether > gasoline, diesel oil > benzene, xylene > ether > hexane. The emulsification rate of HDE-8 EPSs for soybean oil was 31.53 ± 1.02%, which was significantly higher than that of B-2 EPS for soybean oil (7.54 ± 0.34%) [4]. The strong emulsifying properties of extracellular polysaccharides can effectively promote the uniform dispersion of components such as lactobacillus and whey protein in lactobacillus beverages, and help the fat and water phases to better combine in ice cream, thereby enhancing the taste and texture of the products.

### 3.12. Heavy-Metal-Chelating Activity

**Figure 8:**
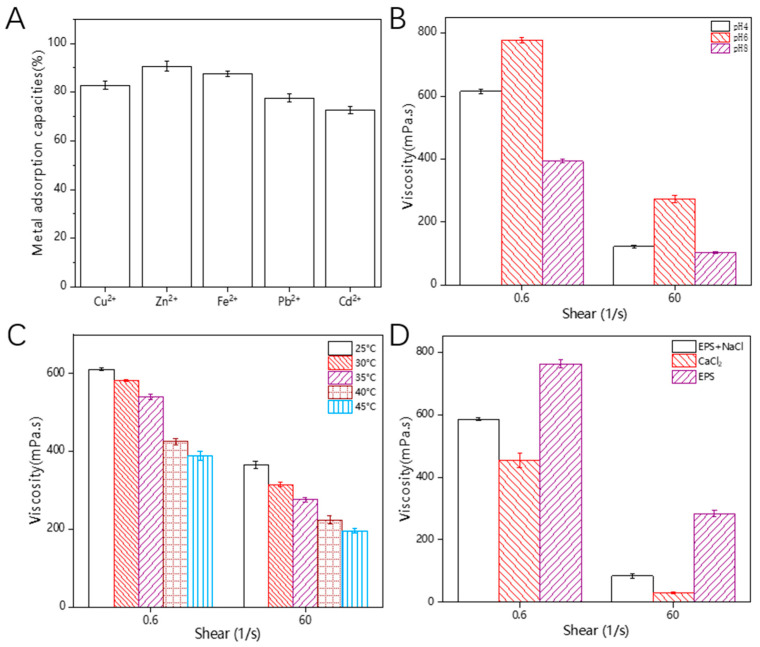
Metal adsorption activity (**A**) and rheological property of EPS ((**B**): pH; (**C**): temperature; (**D**): metal ions).

According to reports, some EPS contain various functional groups such as hydroxyl, ketone, alcohol, amine, thiol, and carboxyl groups. These functional groups allow EPSs to have multiple negative charges, enabling the removal of positively charged heavy metal ions from aqueous solutions [54]. Therefore, EPSs are considered to be promising metal-chelating agents. As shown in Figure 8A, HDE-8 EPSs exhibit a high removal efficiency for Cu^2+^, Zn^2+^, Fe^2+^, Pb^2+^, and Cd^2+^ in solution, with adsorption capacities of 82.84%, 90.69%, 87.56%, 77.62%, and 72.78%, respectively. HDE-8 EPSs exhibit lower adsorption rates for Fe^2+^, Zn^2+^, and Cu^2+^ compared to the previously reported *Bacillus tequilensis* EPS, which demonstrated adsorption rates of Fe^2+^ (94.96%), Zn^2+^ (97.66%), and Cu^2+^ (98.24%) [55]. However, the adsorption rates of the HDE-8 EPS surpass those of the *Pseudoalteromonas* sp. EPS, which recorded adsorption rates of Fe^2+^ (85.00%), Zn^2+^ (58.15%), and Cu^2+^ (52.77%) [56]. The HDE-8 EPS has a good adsorption capacity for heavy metals in water, which is expected to reduce the potential hazards of heavy metals to aquaculture and soil, and contribute to the sustainable development of aquaculture and fruit and vegetable cultivation.

### 3.13. Rheological Property

From Figure 8B–D, it can be observed that the viscosity of the HDE-8 EPS solution decreases with the increasing shear rate. This phenomenon may be attributed to the increased shear rate causing intermolecular interactions within the polysaccharide molecules, such as the formation of chemical bonds like hydrogen bonds, which ultimately affect the decrease in the viscosity of the solution. These interactions occur under relatively static conditions, ultimately resulting in an increased degree of polymerization [12]. In Figure 8B, it can be observed that the EPS solution exhibited the highest viscosity under neutral conditions, while lower viscosities were observed under acidic and alkaline conditions. This result was consistent with previous findings, where acidic or alkaline conditions were found to cause the disruption of hydrogen bonds. This disruption leads to a decrease in the Mw and degradation of EPSs. Consequently, the viscosity of the EPS decreases as a result of this degradation [57,58]. Additionally, Figure 8C demonstrates a decrease in EPS viscosity with an increasing temperature. These phenomena can be attributed to increased molecular motion. It is well-known that when polysaccharides are heated, the extension of polysaccharide chains increases the volume of the molecules. This weakens the intermolecular interaction forces and strengthens the thermal motion between polysaccharide molecular chains, resulting in a decrease in viscosity [59]. This similarity between the observed decrease in viscosity with the increasing temperature and the previously reported decrease in viscosity of EPS-C47 indicates that the viscosity of EPSs exhibits temperature-dependent behavior [60]. Metal ions have an impact on the viscosity of EPS solutions. This can be observed in Figure 8D, where the addition of metal ions leads to a reduction in viscosity. One possible reason for this outcome could be linked to electrostatic repulsion. The charge density of the crosslinked network escalates swiftly upon the addition of a reduced amount of salt ions, resulting in heightened electrostatic repulsion. With the gradual addition of more salt ions, the electrostatic repulsion between charged molecules is shielded, thereby restricting the chain stiffness. The molecular chains begin to curl and twist, causing molecular contraction, which results in reduced viscosity and a disruption of the gel network structure [61,62]. The experimental results show that, compared to the addition of NaCl, the addition of CaCl_2_ leads to a lower viscosity of EPS. This was consistent with previous reports, which indicate that divalent ions (Ca^2+^) have a greater impact on the viscosity of *Dictyophora rubrovolvata* EPSs than monovalent ions (Na^+^) and adding CaCl_2_ can reduce the viscosity of *D. rubrovolvata* EPSs [63]. As a result, EPSs exhibit significant viscoelastic and pseudoplastic behavior, making them suitable as a thickening agent in food. They can be used to increase the viscosity and texture of food, for example, in creams, sauces, ice cream, and jellies. They can also be used as a stabilizer to help maintain the thickness and consistency of food. EPSs have a wide range of applications as a food thickening agent, meeting the needs of different food products.

## 4. Conclusions

In this study, LAB that produces EPSs were isolated from fermented longan juice and identified as *L. mesenteroides* HDE-8. *L. mesenteroides* HDE-8 exhibited a high EPS yield of 25.1 g/L. Through isolation and purification, it was found that the EPSs mainly consist of α-(1→4)- and α-(1→6)-linked *D*-glucose pyranose. The estimated Mw of the EPSs was 1.7 × 10^6^ Da. The EPSs demonstrated a high thermal stability, metal adsorption capacity, and antioxidant activity. Additionally, EPSs had high thermal stability, viscosity, emulsifying properties, and a strong coagulating effect in dairy products, making them suitable as a food additive to improve the texture and mouthfeel of food. These EPSs are suitable for use as a food additive in the dairy industry, such as increasing the viscosity and texture of milk. However, this study only explored the potential of HDE-8 EPSs in food applications, and further detailed research is needed on their application in dairy products.

## Figures and Tables

**Table 1:** EA% of EPSs with organic agents.

Organic Agents	E_24_ (%)	E_72_ (%)
Hexane	3.13 ± 0.06 ^F, a^	6.32 ± 0.10 ^E, b^
Benzene	9.00 ± 0.48 ^D, a^	14.74 ± 0.44 ^D, b^
Xylene	7.46 ± 0.37 ^D, a^	14.27 ± 0.34 ^D, b^
Petroleum ether	16.15 ± 0.54 ^B, a^	25.06 ± 0.26 ^B, b^
Ether	5.07 ± 0.10 ^E, a^	7.60 ± 0.11 ^E, b^
Gasoline	12.97 ± 0.68 ^C, a^	18.79 ± 0.42 ^C, b^
Diesel oil	11.99 ± 0.60 ^C, a^	17.62 ± 0.45 ^C, b^
Soybean oil	21.94 ± 0.69 ^A, a^	31.53 ± 1.02 ^A, b^

Capital letters denote significant differences in data within a column. Lowercase letters indicate significant differences of data in one row.

## Data Availability

Data are contained within the article.
